# *MPRIP::PDGFRB* Fusion Gene: A Rare Case Report of Adult Myeloid/Lymphoid Neoplasm With Eosinophilia and Tyrosine Kinase Gene Fusions

**DOI:** 10.1155/crh/7098722

**Published:** 2025-06-27

**Authors:** Taksin Ukkahad, Tanapun Thamgrang

**Affiliations:** ^1^Medical Education Center, Naradhiwas Rajanagarindra Hospital, Narathiwat, Thailand; ^2^Division of Hematology, Department of Medicine, Phramongkutklao Hospital, Bangkok, Thailand

**Keywords:** *MPRIP*, myeloid/lymphoid neoplasm with eosinophilia and tyrosine kinase gene fusions, *PDGFRB*

## Abstract

Myeloid/lymphoid neoplasms with eosinophilia and tyrosine kinase gene fusions (MLN-TK) represent rare hematological malignancies driven by pathological fusion genes involving tyrosine kinase genes. Among these, rearrangements of the *PDGFRB* gene, particularly the *ETV6::PDGFRB* rearrangement, are frequently observed as pathogenic mutations. Conversely, instances of the *MPRIP::PDGFRB* fusion gene are rarely documented. In this case report, we present a 32-year-old previously healthy Thai male who presented to the hospital with constitutional symptoms and marked splenomegaly. His complete blood count revealed mild anemia, marked leukocytosis with hypereosinophilia, and mild thrombocytopenia. A bone marrow study showed hypercellular marrow with granulocytic hyperplasia extensively involved with eosinophils, without morphological evidence of blasts. Conventional cytogenetics identified a t (5; 17) (q33; p13). Further targeted RNA analysis using next-generation sequencing (NGS) detected a fusion gene involving *MPRIP::PDGFRB*. The patient was diagnosed with myeloid/lymphoid neoplasms with eosinophilia and *MPRIP::PDGFRB* rearrangement in the chronic-phase disease and was initiated on oral imatinib at a daily dose of 100 mg. One month after initiating the treatment, the patient achieved a hematological response consistent with complete response (CR) criteria. Imatinib therapy has been well-tolerated without reported adverse events, and a 1-year molecular assessment confirmed the achievement of complete molecular response (CMR).

## 1. Introduction

Myeloid/lymphoid neoplasms with eosinophilia and tyrosine kinase gene fusions (MLN-TK) constitute a rare group of hematologic malignancies driven by rearrangements involving genes encoding specific tyrosine kinases. This entity was first recognized as an independent disease category in the 2008 World Health Organization (WHO) classification. The recently updated 5^th^ edition of the WHO classification, along with the International Consensus Classification (ICC), has expanded this category to include neoplasms with *PDGFRA, PDGFRB, FGFR1, JAK2,* and *FLT3* rearrangements, as well as *ETV6::ABL1* fusion and other defined tyrosine kinase fusions [1, 2].

In MLN-TK cases, *PDGFRA* rearrangements typically involve cytogenetically cryptic deletion of 4q12, resulting in the *FIP1L1::PDGFRA* fusion. Conversely, *PDGFRB* rearrangements commonly arise from t (5; 12) (q32; p13.2), leading to the *ETV6::PDGFRB* fusion [3]. However, *PDGFRB*-rearranged cases have been associated with more than 40 other fusion partners, such as *PDE4DIP*, *SPECC1*, *TP53BP1*, *WDR48*, and *NTRK3*. Conventional cytogenetic analysis often identifies a translocation at 5q31–33 that leads to the formation of an abnormal *PDGFRB* fusion gene [4].

In this report, we demonstrated a rare tyrosine kinase gene fusion, *MPRIP::PDGFRB* rearrangement, identified in a 32-year-old Thai male patient diagnosed with adult MLN-TK. The correlation of clinical manifestations, morphologic findings, cytogenetic analysis, and molecular studies is crucial for the precise diagnosis of this rare condition.

## 2. Case Presentation

A 32-year-old previously healthy Thai male, without any known medical conditions or current medications, presented to a private hospital and was subsequently referred to our medical center due to loss of appetite and unexplained weight loss of approximately 6 kg over 4 months. He reported experiencing nocturnal fever and night sweats. His symptoms slowly progressed over time. He reported no organ-specific symptoms, apart from intermittent pruritus affecting various areas of his body, including the thighs, trunk, and both arms, without any visible skin manifestations.

The physical examination revealed a low-grade fever of 37.8°C and mild tachycardia (110 beats per minute), mildly pale conjunctivae, and marked splenomegaly extending 6 cm below the left costal margin. Other systemic examinations showed no lymphadenopathy, no hepatomegaly (liver span approximately 10 cm), and normal skin appearance.

**Figure 1:**
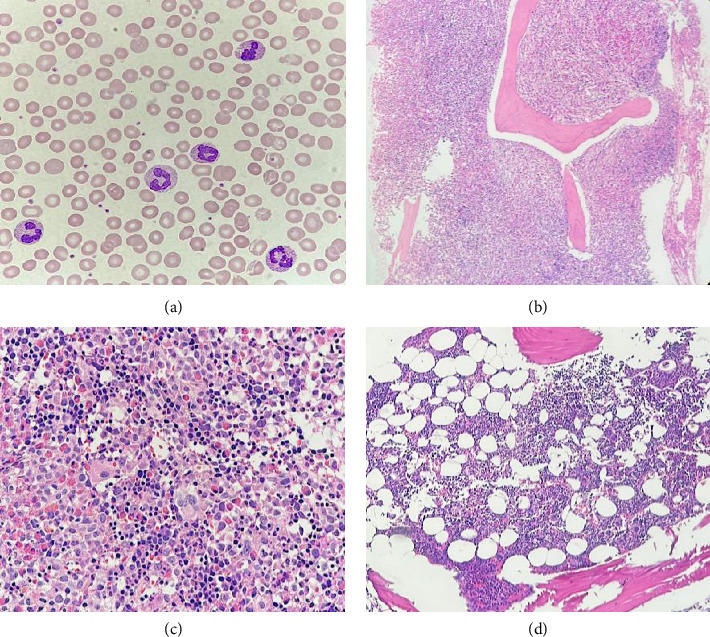
Peripheral blood smear shows normochromic normocytic anemia, leukocytosis with neutrophil predominance, and eosinophilia ((a), 1000x). Bone marrow H&E staining reveals hypercellular marrow with granulocytic hyperplasia, eosinophilia, and dysplastic megakaryocytes ((b), 100x; (c), 400x). Postimatinib therapy, the marrow is normocellular without dysplastic megakaryocytes ((d), 100x).

The initial complete blood count (CBC) revealed hemoglobin (Hb) of 10.3 g/dL, hematocrit (Hct) of 34.1%, mean corpuscular volume (MCV) of 97.7 fL, red cell distribution width (RDW) of 19.9%, and white blood cell (WBC) count of 33.3 × 10^9^/L, with differential showing neutrophils 40%, lymphocytes 17%, monocytes 10%, eosinophil 20%, basophils 2%, promyelocytes 3%, myelocytes 3%, metamyelocytes 5% and platelet count 80 × 10^9^/L. The peripheral blood smear revealed normochromic normocytic anemia without anisocytosis or poikilocytosis, marked leukocytosis with neutrophil predominance, significant eosinophilia without dysplastic features, and mildly decreased platelet count with normal platelet staining (Figure 1(a)). His blood chemistry profile, including electrolytes, kidney function, and liver function tests, were within normal limits. However, serum lactate dehydrogenase (LDH) was elevated at 321 U/L (normal value < 225 U/L). Ultrasonography of the upper abdomen revealed normal size and parenchymal echogenicity of the liver. The spleen was moderately enlarged, measuring approximately 16.3 × 7.4 cm, without focal masses, and no other abnormalities were noted.

The initial bone marrow study was conducted for diagnostic purposes. The bone marrow biopsy exhibited features consistent with myeloproliferative disease, characterized by marked hypercellularity (95% cellular marrow) and an increased myeloid-to-erythroid ratio of 10:1. There was prominent granulocytic hyperplasia with eosinophilia involving 80% of the marrow. Megakaryocytes were moderately decreased and showed clustering, focal small-sized forms, and hypolobated morphology. There was no morphological evidence of increased blast cells. In addition, moderately increased reticulin fibrosis (MF Grade 2) was observed (Figures 1(b) and 1(c)).

**Figure 2:**
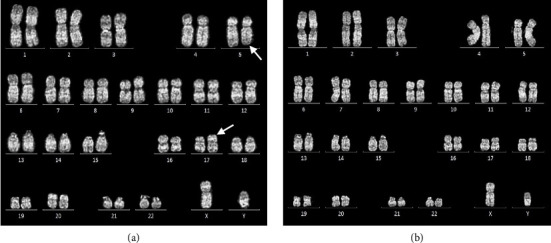
Q-Banded karyotype analysis shows 46,XY, t (5; 17) (q33; p13) [10] indicated by the arrows (a). Following imatinib therapy, the karyotype reveals a 46, XY [18], normal male karyotype (b).

**Figure 3:**
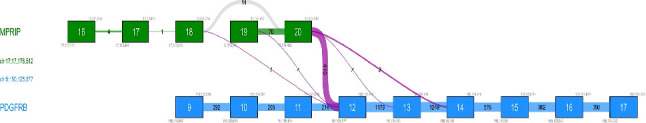
RNA-targeted fusion gene sequencing for myeloid neoplasms by next-generation sequencing (NGS) identified an *MPRIP::PDGFRB* fusion gene.

Conventional chromosome analysis identified a t (5; 17) (q33; p13) in 10 out of 20 metaphases analyzed (Figure 2(a)). Bone marrow RT-PCR for the *BCR::ABL1* gene yielded negative results. To further investigate, molecular cytogenetic analysis was performed. DNA-based targeted gene sequencing of peripheral blood using a next-generation sequencing (NGS) panel for myeloid neoplasms detected no pathogenic or likely pathogenic mutations. However, RNA-based targeted fusion gene analysis using an NGS panel covering 54 myeloid-associated pathogenic genes (*ABL1, AFDN, ALK, BCL11B, BCOR, BCR, CBFB, CEP43, CNTRL, DEK, ELL, EML1, ETV6, FGFR1, FGFR2, FIP1L1, FLT3, GOLGB1, IQCB1, JAK2, KMT2A, LYN, MECOM, MIX23, MLLT1, MLLT3, MLLT10, MRTFA, MYH11, NDE1, NPM1, NTRK3, NUMA1, NUP98, PCM1, PDGFRA, PDGFRB, PML, PRKG2, PSMD2, RANBP2, RARA, RBM15, RCSD1, RET, RUNX1, RUNX1T1, SPTBN1, STAT5B, TRIP11, WT1, ZBTB16, ZMYM2,* and *ZNF384*) identified a novel *MPRIP::PDGFRB* fusion gene (Figure 3).

The clinical features, supported by laboratory, histologic, and molecular cytogenetic findings, confirmed the diagnosis of myeloid/lymphoid neoplasm with eosinophilia and *MPRIP::PDGFRB* rearrangement in the patient (chronic-phase disease according to MLN International Working Group classification) [5]. Treatment commenced with the first-generation tyrosine kinase inhibitor (TKI), imatinib, administered orally at a daily dose of 100 mg. Following 1 month of imatinib therapy, the patient's condition showed significant improvement, characterized by a notable increase in appetite and a weight gain of 4 kg. There were no reports of night sweats or pruritic sensations, and the patient did not experience any imatinib-related side effects. Physical examination revealed no pale conjunctivae, a spleen of normal size below the left costal margin, and a normal liver span. CBC revealed Hb of 12.6 g/dL, Hct of 40.2%, MCV of 94.2 fL, RDW of 16.3%, and WBC count of 4.4 × 10^9^/L, with differential showing neutrophils 39.6%, lymphocytes 48.5%, monocytes 6.1%, basophils 1%, eosinophil 4.8%, and platelet count 179 × 10^9^/L. Three months after initiating imatinib therapy, serial ultrasonographic assessment of the upper abdomen revealed normal liver and spleen size with unremarkable parenchymal echogenicity. Based on the hematologic response assessed according to the MLN International Working Group response criteria, the patient met the complete response (CR) criteria. The patient has been receiving 100 mg of imatinib orally to date without evidence of clinical relapse. After 1 year of imatinib therapy, a bone marrow study and molecular analysis were conducted. Bone marrow biopsy revealed normocellular marrow with multilineage maturation, adequate megakaryocytes, and normal morphology, with no evidence of eosinophilia, myeloproliferative neoplasm (MPN), or increased blasts Figure 1(d)). Conventional cytogenetic analysis demonstrated 46, XY [18], normal male karyotype (Figure 2(b)), and RNA-targeted gene fusion for myeloid neoplasms was negative for *MPRIP::PDGFRB*. However, the *OAZ1::MLLT1* fusion gene was detected, aligning with complete molecular response (CMR) criteria at the 1-year evaluation. To confirm the absence of the pathogenic fusion gene, a second molecular analysis was performed. Fluorescence in situ hybridization (FISH) targeting the *PDGFRB* gene demonstrated a negative result, further supporting the presence of molecular remission.

## 3. Discussion

We discussed a case involving a 32-year-old Thai male who presented to the hospital with constitutional symptoms and splenomegaly lasting for six months. An initial CBC revealed mild anemia, leukocytosis with marked eosinophilia, and mild thrombocytopenia. A bone marrow study was consistent with myeloproliferative disease, showing hypercellular marrow, granulocytic hyperplasia with extensive involvement of eosinophils, and no histologic evidence of increased blasts. Conventional cytogenetic analysis revealed a t (5; 17) (q33; p13). Targeted RNA analysis using NGS detected a fusion gene involving *MPRIP::PDGFRB*. The patient was diagnosed with a myeloid/lymphoid neoplasm with eosinophilia and *MPRIP::PDGFRB* rearrangement, indicative of chronic-phase disease. Initial treatment with imatinib at a daily oral dose of 100 mg induced a complete hematologic response within 1 month, with no reported side effects, and achieved a CMR after 1 year of therapy.

According to the German Registry for Disorders of Eosinophils and Mast Cells (GREM) registry–based cohort study [6], this case is consistent with the typical age of presentation, commonly seen in middle-aged individuals, with a median age of 45.5 years (range: 19–70 years). In addition, the condition has a higher prevalence in males than in females, with a striking male predominance (81%–91% male) [7]. Clinical features frequently include myelodysplastic (MDS)/MPN–type characteristics with marked eosinophilia, although eosinophilia is not universal. These features often resemble chronic myelomonocytic leukemia (CMML) with eosinophilia [8, 9]. Other common symptoms include constitutional symptoms and splenomegaly. Most cases present in the chronic phase of the disease rather than in the accelerated phase (AP) or with extramedullary disease (EMD).

While *PDGFRB* rearrangement is the second most identified abnormality in MLN-TK [5], the *MPRIP::PDGFRB* rearrangement is rarely documented. The *MPRIP::PDGFRB* rearrangement was first reported in 2015 in an 80-year-old male with chromosome 46, XY, t (5; 17) (q33; p11), who presented with chronic-phase MLN-TK, splenomegaly, and blood hypereosinophilia, but no monocytosis [10]. The patient responded to imatinib 100 mg/day and achieved a complete cytogenetic remission within 6 months. Another case report described a 37 year-old male who also had hypereosinophilia but with no reported monocyte percentage or spleen size [11]. This patient also showed a good response to imatinib 100 mg/day and achieved a CMR within 6 months.

Notably, although hypereosinophilia is frequently reported in patients with *FIP1L1::PDGFRA* and *ETV6::ABL1* fusion genes [12, 13], only 50%–80% of patients with *PDGFRB* rearrangement are reported to have hypereosinophilia. Hypereosinophilia was also observed in this patient and other reported cases with the *MPRIP::PDGFRB* rearrangement.

The detection of the *OAZ1::MLLT1* fusion gene was unexpectedly found during follow-up RNA-targeted fusion gene sequencing for myeloid neoplasms by NGS. However, there are no previous reports regarding the significance of this fusion gene in myeloid neoplasms. This could be due to false-positive results from RNA fusion detection by NGS [14]. This finding is most likely a sequencing artifact rather than a true pathogenic driver.

Previous studies have shown that patients with MLN-TK with *PDGFRB* rearrangements generally respond well to TKIs [15]. Patients who achieve complete cytogenetic response (CCyR) or CMR following imatinib therapy typically do not experience relapse or progression to the blastic phase. The 1-year overall survival rate was 90%, with a 6-year progression-free survival rate of 88% observed in imatinib-treated patients. These patients typically achieve a first hematologic response within the first 3 months of induction therapy with the first-generation TKI, imatinib, at an initial daily dose of 100 mg. A comprehensive hematologic response, characterized by the restoration of peripheral blood counts and normalization of eosinophilia, is typically achieved within 1 month, while CCyR is attained within 3 months in most patients.

Although a daily dose of 400 mg of imatinib is generally used as the standard treatment for a chronic-phase disease, with a good response rate as recommended by the NCCN guidelines [16], this report demonstrates that a 100 mg induction dose of imatinib can also lead to a favorable outcome. Previous cases of *MPRIP::PDGFRB* rearrangement have shown that a 100 mg/day dose of imatinib produced a positive response.

The possibility of stopping imatinib in patients with MLN-TK with *PDGFRB* rearrangement has not been assessed. Currently, there are no established criteria to determine which patients may be appropriate candidates for discontinuation of imatinib, and therefore discontinuation is not recommended outside of clinical trial settings due to the risk of relapse [17].

## 4. Conclusion

MLN-TK are rare hematologic malignancies with diverse clinical presentations. Molecular cytogenetic studies frequently identify *PDGFRB* gene rearrangements as pathogenic mutations; however, the *MPRIP::PDGFRB* fusion gene is rarely documented and typically presents with features characteristic of *PDGFRB* rearrangement. First-generation TKIs, imatinib, at a daily dose of 100 mg, have demonstrated efficacy and are well-tolerated.

## Data Availability

Data are available on request from the authors.
